# Personalized Optimal Fat Intake for Glycemic Health: Evidence of a U‐Shaped Association and Heterogeneity From NHANES

**DOI:** 10.1155/jdr/3991280

**Published:** 2026-03-12

**Authors:** Xiaoyan Huang, Miaohui Wu, Baoliang Huang

**Affiliations:** ^1^ Department of Endocrinology, Fujian Medical University Affiliated First Quanzhou Hospital, Quanzhou, Fujian, China; ^2^ Department of Pharmacy, Jinjiang Municipal Hospital, Quanzhou, Fujian, China; ^3^ Department of Intensive Care Unit, Fujian Medical University Affiliated First Quanzhou Hospital, Quanzhou, Fujian, China

**Keywords:** dietary fat, glycemic control, NHANES, nonlinear association, personalized nutrition

## Abstract

**Background:**

The optimal dietary fat intake for glycemic control is debated. We investigated the dose‐response relationship between the fat energy ratio and HbA1c, determined an optimal level, and assessed effect modification by age and diabetes status.

**Methods:**

This cross‐sectional study included 6639 U.S. adults from NHANES 2017–2020. The fat energy ratio was derived from two 24‐h dietary recalls. Associations with HbA1c were assessed using restricted cubic splines and multivariable regression, adjusting for sociodemographic, lifestyle, clinical, and dietary covariates.

**Results:**

A significant nonlinear, U‐shaped association was identified (P − nonlinearity < 0.01). The optimal fat intake for minimal HbA1c was 36.0% (95% CI: 32.1–38.2). Stratified analyses revealed effect modification: the optimal ratio was higher in middle‐aged (45–64 years: 36.9%) versus younger (18–44 years: 32.2%) and older adults (≥ 65 years: 32.7%), and in individuals with diabetes (38.0%) who exhibited a pronounced U‐shaped curve, unlike the flat association seen in those without diabetes.

**Conclusion:**

Glycemic control exhibits a U‐shaped relationship with dietary fat, with an overall optimum at 36.0% of energy. The ideal intake is context‐dependent, being higher in middle‐aged and diabetic populations, supporting personalized dietary guidelines over universal fat intake targets.

## 1. Background

Dietary fat intake has long been a central focus in nutritional epidemiology and public health guidelines for metabolic disease prevention. For decades, the primary emphasis was on reducing total fat intake, leading to the widespread promotion of low‐fat diets [1–3]. However, this paradigm is being reevaluated, as the optimal level of total fat intake, rather than its mere reduction, constitutes the central controversy in contemporary research. This is particularly evident in the polarized debate between very‐low‐fat and high‐fat (e.g., ketogenic) dietary patterns, each claiming benefits for metabolic health [4–6].

The specific relationship between the proportions of energy derived from dietary fat (fat energy ratio) and medium‐term glycemic control, as measured by glycated hemoglobin (HbA1c), remains inadequately characterized. Many prior analyses have assumed a linear relationship between fat intake and health outcomes, potentially obscuring complex, nonlinear associations [7, 8]. However, evidence regarding the impact of specific macronutrient proportions on diabetes‐related markers, such as glycemic control and insulin resistance, remains fragmented. Divergent findings have been reported, with some studies indicating potential benefits of certain dietary patterns, whereas others highlight risks, leading to a lack of unified conclusions regarding optimal intake levels [9–11]. Furthermore, a critical gap exists in understanding how this relationship might vary across key demographic and clinical subgroups, such as by age and diabetes status. Additionally, nutritional guidelines for specific populations, such as those with diabetes, often recommend a range for fat intake (e.g., 20%–35% of total energy), yet the empirical evidence defining the precise value within this range that optimizes glycemic outcomes is often lacking [12, 13].

This scientific uncertainty persists against a backdrop of intense public and academic debate. To address these evidence gaps, this study utilized data from the National Health and Nutrition Examination Survey (NHANES) with three primary objectives: first, to elucidate the shape of the dose‐response relationship between total fat energy ratio and HbA1c; second, to identify an optimal intake level associated with the most favorable glycemic profile; and third, to investigate effect modification by age and diabetes status. The findings are aimed at providing an empirical basis for moving beyond simplistic dietary recommendations toward personalized nutrition.

## 2. Methods

### 2.1. Study Design and Population

This cross‐sectional analysis utilized data from the NHANES 2017–2020, a nationally representative survey of the noninstitutionalized U.S. population conducted by the National Center for Health Statistics (NCHS). NHANES employs a stratified, multistage probability sampling design. Publicly available data were accessed via https://wwwn.cdc.gov/nchs/nhanes/. The study included participants aged 18 years or older. Exclusion criteria were age 17 years or younger, or missing data on daily fat intake, total energy intake, or HbA1c.

### 2.2. Participant Selection Process

**Figure 1:**
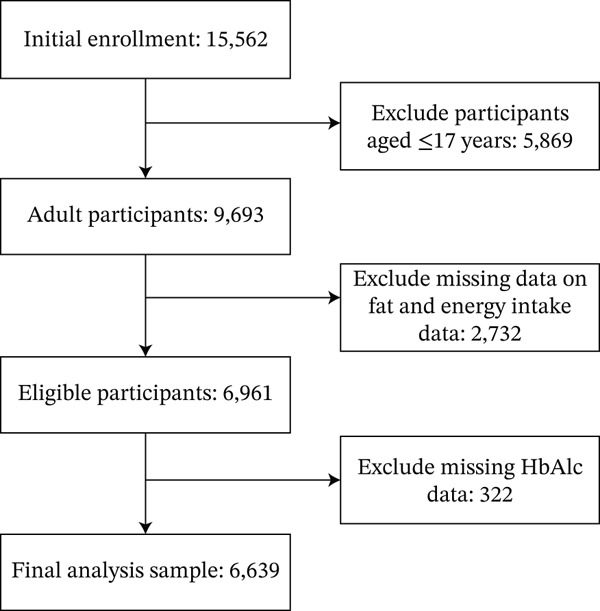
Participant selection flowchart.

A total of 15,562 participants were initially enrolled in this study. Among these, 5869 participants aged ≤ 17 years were excluded, yielding 9693 adult participants. Subsequently, 2732 participants with missing data on daily fat and energy intake were excluded, leaving 6961 eligible participants. Finally, 322 participants with missing HbA1c data were excluded, and the final analysis sample consisted of 6639 participants (Figure 1).

### 2.3. Variable Definitions

#### 2.3.1. Independent variable

The independent variable, the fat energy ratio (%), was defined as the proportion of total daily energy derived from dietary fat. This ratio was calculated by first determining the energy contribution of fat (kcal), obtained by multiplying the fat intake (g) by its energy conversion factor (9 kcal/g), and then dividing this value by the total daily energy intake and multiplying by 100. Dietary intake data were collected through two 24‐h dietary recalls: The initial recall was conducted in‐person at the mobile examination center (MEC), followed by a telephone‐based recall 3–10 days later to account for day‐to‐day variability. Fat content and energy values of all consumed foods and beverages were calculated using the USDA Food and Nutrient Database for Dietary Studies (FNDDS). For participants with complete data from both recalls, the mean fat energy ratio across the 2 days was used. To ensure representativeness of the U.S. population and correct for the complex sampling design of NHANES, dietary weights (WTDRD1PP for the first recall and WTDRD2PP for the second recall) were applied during data analysis.

#### 2.3.2. Dependent Variable

HbA1c (%) was analyzed from whole blood specimens using high‐performance liquid chromatography at the University of Missouri‐Columbia.

#### 2.3.3. Covariates

Adjusted covariates encompassed demographic factors, lifestyle behaviors, clinical conditions, and dietary composition, with specific units/categories as follows:

• Demographic factors: Age (years); sex: male, female; race/ethnicity: Mexican American, other Hispanic, non‐Hispanic White, non‐Hispanic Black, and other race (including multiracial); education level: less than high school, high school graduate, and more than high school; marital status: married, nonmarried (including single, divorced, and widowed); and income level: low income (≤ 130% of the U.S. federal poverty line), middle income (131%–350% of the federal poverty line), and high income (> 350% of the federal poverty line).
• Lifestyle behaviors: Smoking status: current smoker (smokes ≥ 1 cigarette per day), former smoker (previously smoked but not current), and never smoker (never smoked); alcohol use: current drinker (consumes alcohol ≥ 1 time per month), former drinker (previously drank but not current), and never drinker (never drank); and physical activity: low (no moderate/vigorous physical activity per week), moderate (150–299 min of moderate activity or 75–149 min of vigorous activity per week), and high (≥ 300 min of moderate activity or ≥ 150 min of vigorous activity per week).
• Clinical conditions: Body mass index (BMI, kg/m^2^); hypertension: yes (self‐reported diagnosis, measured blood pressure ≥ 140/90 mmHg, or antihypertensive medication use), no; hypercholesterolemia: yes (self‐reported history, total cholesterol ≥ 240 mg/dL [6.21 mmol/L], or lipid‐lowering drug use), no; diabetes: yes (self‐reported diagnosis, fasting glucose ≥ 126 mg/dL [7.0 mmol/L], HbA1c ≥ 6.5%, or use of glucose‐lowering agents), no; fasting blood glucose (mmol/L, mg/dL); and insulin (pmol/L).
• Dietary composition: Carbohydrate energy ratio (%); protein energy ratio (%); and total energy intake (kcal/day).

All analyses incorporated appropriate examination (WTMEC4YR) and dietary weights to account for complex sampling and nonresponse.

### 2.4. Statistical Analysis

Statistical analyses were performed using R software (Version 4.5.1). Continuous variables were summarized as mean ± standard deviation (SD) if they approximately followed a normal distribution (assessed by the Shapiro–Wilk test, *p* > 0.05), or as median (interquartile range) if skewed (Shapiro–Wilk test, *p* ≤ 0.05). It is important to note that true biological variables do not adhere to a strict normal distribution (as negative values are physiologically implausible); thus, the criterion of “approximate normality” is widely adopted in epidemiological research. Parametric tests (e.g., one‐way Analysis of Variance [ANOVA], linear regression) remain robust for such approximately normal variables, especially with large sample sizes. Categorical variables were expressed as percentages. Group differences across fat intake quartiles were assessed using one‐way ANOVA or Kruskal–Wallis tests for continuous variables, and Pearson′s chi‐square tests for categorical variables.

Hierarchical multiple linear regression models were designed to sequentially quantify the confounding/mediating effects of different covariate groups, assessing the relationship between fat energy ratio and HbA1c as follows: Model 1 controlled for sociodemographic variables (age, sex, and race) to exclude the influence of innate, nonmodifiable factors and establish a baseline association; Model 2 added BMI and lifestyle factors (smoking, alcohol use, and physical activity) to evaluate the mediating role of modifiable behaviors in the fat‐HbA1c relationship; Model 3 further included clinical comorbidities (hypertension and diabetes) and dietary components (carbohydrate and protein energy ratios, total energy) to account for residual confounding from clinical status and macronutrient balance, yielding the independent association of fat intake with HbA1c. A sensitivity analysis excluded participants with diabetes. Nonlinear associations were explored using restricted cubic splines (RCS) with three knots; the optimal fat intake level was determined by identifying the nadir of the fitted curve. Bootstrap resampling with 1000 replications validated the robustness of this estimate. Confidence intervals (CIs) were used to quantify the uncertainty of parameter estimates. The standard error (SE) was reported to indicate the precision of regression coefficients. Subgroup analyses were performed by age and diabetes status. A two‐sided *p* value < 0.05 indicated statistical significance.

## 3. Results

### 3.1. Baseline Characteristics of the Study Population

Table 1 presents the baseline characteristics of the 6639 study participants stratified by quartiles (Q) of the fat energy ratio. Participants in the highest quartile of fat intake (Q4) were slightly older and had a higher BMI compared with those in the lowest quartile (Q1) (50.70 vs. 49.12 years, *p* = 0.017; 31.28 vs. 29.15 kg/m^2^, *p* < 0.001, respectively). The distribution of sex, race, education level, and income also differed significantly across quartiles (all *p* < 0.001). Notably, the proportion of non‐Hispanic White individuals and those with education beyond high school increased with higher fat intake.

**Table 1:** Baseline characteristics by quartiles of fat energy ratio.

Variables	*T* *o* *t* *a* *l* = 6639	Quartiles of fat energy ratio				*p* value
		Q1 *N* = 1647	Q2 *N* = 1673	Q3 *N* = 1669	Q4 *N* = 1650	
**Age(years)**	49.47 ± 18.22	49.12 ± 17.58	48.97 ± 18.20	49.13 ± 18.55	50.70 ± 18.12	0.017
**Sex (%)**						0.001
male	3174 (47.80%)	856 (51.97%)	768 (45.90%)	779 (46.67%)	771 (46.73%)	
Female	3465 (52.20%)	791 (48.03%)	905 (54.10%)	890 (53.33%)	879 (53.27%)	
**Race (%)**						< 0.001
Mexican American	774 (11.66%)	231 (14.03%)	206 (12.31%)	168 (10.07%)	169 (10.24%)	
Other Hispanic	665 (10.02%)	242 (14.70%)	177 (10.58%)	141 (8.45%)	105 (6.36%)	
Non‐Hispanic White	2418 (36.42%)	489 (29.69%)	594 (35.51%)	678 (40.62%)	657 (39.82%)	
Non‐Hispanic Black	1793 (27.01%)	344 (20.89%)	462 (27.62%)	462 (27.68%)	525 (31.82%)	
Other Race (Including Multiracial)	989 (14.90%)	341 (20.70%)	234 (13.99%)	220 (13.18%)	194 (11.76%)	
**Education level (%)**						< 0.001
< High school	1076 (16.21%)	360 (21.86%)	272 (16.26%)	246 (14.74%)	198 (12.00%)	
High school	1574 (23.71%)	377 (22.89%)	397 (23.73%)	409 (24.51%)	391 (23.70%)	
> High school	3989 (60.08%)	911 (55.31%)	1005 (60.07%)	1015 (60.81%)	1058 (64.12%)	
**Married (%)**	3864 (58.20%)	988 (59.99%)	1005 (60.07%)	942 (56.44%)	929 (56.30%)	0.273
**Income level (%)**						< 0.001
Low income	1880 (28.32%)	524 (31.82%)	469 (28.03%)	464 (27.80%)	423 (25.64%)	
Middle income	2563 (38.61%)	638 (38.74%)	632 (37.78%)	679 (40.68%)	614 (37.21%)	
High income	2196 (33.08%)	486 (29.51%)	572 (34.19%)	526 (31.52%)	612 (37.09%)	
**Physical activity (%)**						0.301
Low	3731 (56.20%)	930 (56.47%)	928 (55.47%)	937 (56.14%)	936 (56.73%)	
Moderate	1348 (20.30%)	321 (19.49%)	323 (19.31%)	342 (20.49%)	362 (21.94%)	
High	1560 (23.50%)	400 (24.29%)	422 (25.22%)	387 (23.19%)	351 (21.27%)	
**BMI (kg/m** ^**2**^ **)**	30.14 ± 7.63	29.15 ± 7.23	29.91 ± 7.60	30.22 ± 7.64	31.28 ± 7.88	< 0.001
**Current smokers (%)**	2819 (42.46%)	807 (49.00%)	729 (43.57%)	679 (40.68%)	604 (36.61%)	< 0.001
**Alcohol consumption (%)**	5996 (90.31%)	1446 (87.80%)	1499 (89.60%)	1544 (92.51%)	1507 (91.33%)	< 0.001
**Hypertension (%)**	2496 (37.60%)	572 (34.73%)	609 (36.40%)	641 (38.41%)	674 (40.85%)	0.001
**Hypercholesterolemia (%)**	3114 (46.90%)	746 (45.29%)	766 (45.79%)	786 (47.09%)	816 (49.45%)	0.091
**Diabetes (%)**	1248 (18.80%)	283 (17.18%)	264 (15.78%)	305 (18.27%)	396 (24.00%)	< 0.001
**Carbohydrates (% of energy)**	46.62 ± 9.75	54.89 ± 10.06	49.18 ± 6.46	44.79 ± 5.68	37.63 ± 6.87	< 0.001
**Protein (% of energy)**	15.66 ± 4.49	15.38 ± 5.30	15.59 ± 4.22	15.56 ± 3.96	16.13 ± 4.34	< 0.001
**Fat (% of energy)**	36.50 ± 7.76	26.57 ± 4.20	34.18 ± 1.54	39.08 ± 1.41	46.14 ± 4.11	< 0.001
**Total energy intake (kcal/day)**	2028.21 ± 836.27	1880.08 ± 816.87	2025.32 ± 807.40	2117.96 ± 868.99	2088.22 ± 831.04	< 0.001
**Fasting blood glucose (mmol/L)**	6.26 ± 2.06	6.20 ± 2.08	6.17 ± 1.95	6.28 ± 2.12	6.39 ± 2.11	0.145
**Insulin (pmol/L)**	60.54 (37.38,99.14)	55.80 (34.98, 90.96)	61.56 (37.89,100.98)	59.28 (38.40,102.12)	66.00 (39.36,108.00)	0.003
**HbA1c (%)**	5.83 ± 1.11	5.82 ± 1.16	5.76 ± 1.00	5.80 ± 1.03	5.96 ± 1.22	< 0.001

*Note:* Continuous variables are presented as mean ± standard deviation (SD) or median (interquartile range) as appropriate; categorical variables are presented as *n* (%).

Significant differences were also observed in several clinical and behavioral factors. The prevalence of current smoking was highest in Q1 (49.0%) and decreased across quartiles to Q4 (36.6%, *p* < 0.001). Conversely, the prevalence of alcohol consumption, hypertension, and diabetes was lowest in Q1 and increased significantly across quartiles (e.g., diabetes: Q1 = 17.2*%* vs. Q4 = 23.8*%*, *p* < 0.001). Correspondingly, the mean dietary fat contribution increased from 26.57% in Q1 to 46.14% in Q4, accompanied by a reciprocal decrease in carbohydrate intake. Although fasting blood glucose did not differ across groups, both insulin levels and HbA1c were significantly higher in the highest quartile of fat intake (HbA1c: Q1 = 5.82*%* vs. Q4 = 5.96*%*, *p* < 0.001).

### 3.2. Association of Fat Intake With HbA1c

The associations between dietary fat intake and HbA1c levels, derived from hierarchical multiple linear regression analyses, are presented in Table 2. The unadjusted model revealed a significant positive association between fat energy ratio and HbA1c (*B* = 5.514, SE = 0.116, *p* < 0.001). In the main analysis involving the total population, the association between the fat energy ratio and HbA1c demonstrated a dynamic pattern across sequentially adjusted models. A significant positive association was observed in the initial model adjusted for sociodemographic factors (Model 1: *B* = 0.689, SE = 0.304, *p* = 0.023). However, this association was attenuated and became nonsignificant after further adjustment for BMI and lifestyle factors (Model 2: *B* = 0.344, SE = 0.299, *p* = 0.249). All core conclusions regarding the linear association between fat energy ratio and HbA1c are based on the fully adjusted Model 3. In this model, which included clinical comorbidities and other dietary components, the fat energy ratio was again significantly and positively associated with HbA1c levels (*B* = 0.790, SE = 0.315, *p* = 0.012), reflecting the independent effect of fat intake after controlling for all key confounders.

**Table 2:** Association of dietary fat intake with HbA1c: Main and sensitivity analyses.

Variable	Main analysis (total population)		Sensitivity analysis (nondiabetic population)	
	B (95% CIs)	*p* value	B (95% CIs)	*p* value
**Unadjusted Model**	5.514 (5.287–5.741)	< 0.001	0.423 (0.188–0.658)	< 0.001
**Model 1**				
Fat energy ratio (%)	0.689 (0.092–1.286)	0.023	0.307 (0.089–0.525)	0.006
**Model 2**				
Fat energy ratio (%)	0.344 (−0.244–0.932)	0.249	0.182 (−0.030–0.394)	0.091
**Model 3**				
Age (years)	0.006 (0.004–0.008)	<0.001	0.010 (0.008–0.012)	< 0.001
BMI (kg/m^2^)	0.014 (0.008–0.020)	<0.001	0.013 (0.011–0.015)	< 0.001
Diabetes (Yes)	1.769 (1.671–1.867)	<0.001	NA	NA
Smoking (Yes)	0.105 (0.027–0.183)	0.008	0.070 (0.035–0.105)	< 0.001
Carbohydrate energy ratio (%)	0.669 (0.100–1.238)	0.023	0.498 (0.241–0.755)	< 0.001
Protein energy ratio (%)	0.173 (−0.841–1.187)	0.738	0.522 (0.070–0.974)	0.025
Fat energy ratio (%)	0.790 (0.174–1.406)	0.012	0.529 (0.251–0.807)	< 0.001

*Note:* Hierarchical models were adjusted for Model 1: sociodemographics (sex, age, race); Model 2: Model 1 + lifestyle (BMI, smoking, alcohol, physical activity); Model 3 (fully adjusted): Model 2 + clinical factors (hypertension, diabetes) and dietary composition (carbohydrate, protein, total energy). The sensitivity analysis applied all models to the nondiabetic subgroup.

To test the robustness of this finding, a sensitivity analysis was performed by restricting the cohort to nondiabetic participants. A consistent but more pronounced pattern was observed. The positive association was significant in Model 1 (*B* = 0.307, SE = 0.111, *p* = 0.006), attenuated in Model 2 (*B* = 0.182, SE = 0.108, *p* = 0.091), and strongly significant in the fully adjusted Model 3 (*B* = 0.529, SE = 0.142, *p* < 0.001).

In the final model of the main analysis, other factors significantly associated with higher HbA1c included older age, higher BMI, the presence of diabetes, smoking, and a higher carbohydrate energy ratio. In the nondiabetic subgroup, in addition to age, BMI, and smoking, both higher carbohydrate and higher protein energy ratios were also significantly associated with increased HbA1c.

### 3.3. Nonlinear Association and Optimal Level

**Figure 2:**
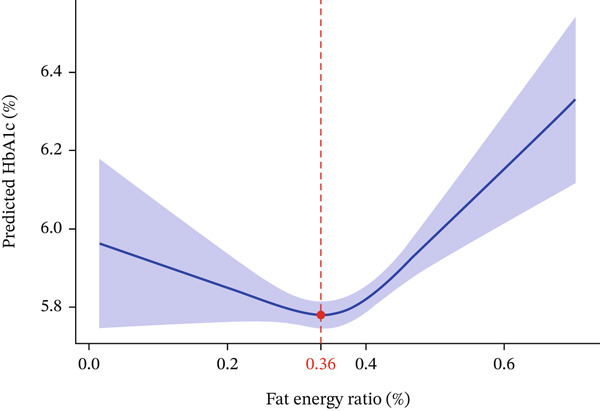
RCS of fat energy ratio and HbA1c.

The RCS analysis revealed a significant nonlinear dose–response relationship between fat intake ratio and HbA1c levels (*p* = 0.0012; Figure 2). Specifically, the association followed a U‐shaped pattern, wherein HbA1c levels increased at both the lower and higher extremes of fat intake. Notably, the curvature analysis indicated a steeper increase in HbA1c at higher fat intake levels compared with the more gradual rise observed at lower intakes.

Further examination of the spline curve identified an optimal fat intake ratio of 36.0%, which corresponded to the minimal predicted HbA1c level. This estimate was further validated by bootstrap resampling analysis (1000 iterations), yielding a median optimal fat ratio of 36.0% (95% CI: 32.1%–38.2%) with low variability (SD = 1.1*%*), underscoring the precision of the estimation. In the study population, fat intake ratios ranged widely from 1.5% to 70.3%, with a mean of 36.5% (SD = 8.3*%*). The interquartile range spanned from 26.2% to 47.6%, indicating that the model‐derived optimal value of 36.0% aligned closely with both the sample mean and the central range of empirically observed intakes.

To further validate the robustness of the primary findings derived from RCS, a quadratic regression model was employed as a supplementary validation approach on the identical fully adjusted dataset. Comparisons between the supplementary quadratic regression and primary RCS results are summarized in Table 3.

**Table 3:** Comparison of optimal dietary fat intake estimates derived from RCS and quadratic regression models.

Group	Analytical method	Test for nonlinearity (*p* value)	Optimal fat intake, % (95% CI)
Total population	RCS	0.0012	36.0 (32.1–38.2)
	Quadratic regression	< 0.001	37.2 (32.8–39.5)
With diabetes	RCS	< 0.001	38.0 (35.7–40.2)
	Quadratic regression	< 0.001	39.5 (36.3–41.8)
Without diabetes	RCS	0.68	Flat association
	Quadratic regression	0.65	Nonsignificant

As illustrated in Table 3, the supplementary quadratic regression yielded results generally consistent with those of the primary RCS analysis: the quadratic term was statistically significant in the total population (*p* < 0.001) and the subgroup of participants with diabetes (*p* < 0.001), whereas it was nonsignificant in the nondiabetic subgroup *(* *p* = 0.65). The turning points estimated by quadratic regression were 37.2% (95% CI: 32.8%–39.5%) in the total population and 39.5% (95% CI: 36.3%–41.8%) in participants with diabetes, with absolute differences of 1.2% and 1.5% relative to the primary RCS‐derived estimates, respectively. These minor discrepancies fall within a reasonable range of methodological variation, confirming that the two approaches yield essentially consistent results.

### 3.4. Stratified Analysis of Optimal Fat Intake by Age

Given the potential for age to modify metabolic responses to dietary intake, we conducted an age‐stratified analysis to ascertain whether the overall U‐shaped association concealed age‐specific optimal fat intake levels.

**Figure 3:**
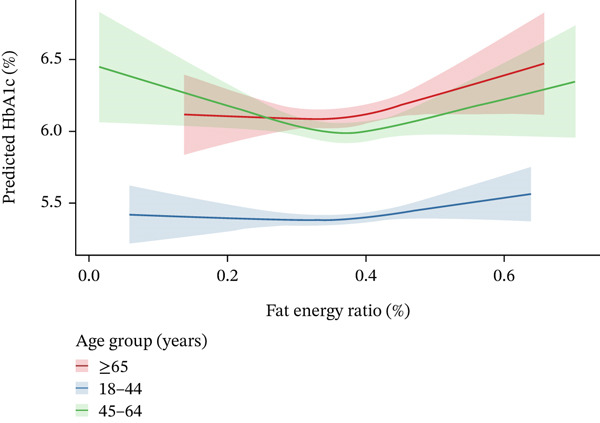
Association between dietary fat energy ratio and HbA1c stratified by age.

Age‐stratified analysis revealed distinct optimal fat intake percentages for minimizing HbA1c across groups (Figure 3): 32.2% in 18–44 years, 36.9% in 45–64 years, and 32.7% in ≥ 65 years. ANOVA demonstrated significant differences in these optimal percentages among age groups (*F* = 21.34, *p* < 0.001). Tukey′s Honest Significant Difference post hoc test further showed the 45–64 years group had a significantly higher optimal fat intake percentage comparedwith both the 18–44 years (*p* < 0.001) and ≥ 65 years (*p* < 0.001) groups, whereas no significant difference existed between 18–44 and ≥ 65 years (*p* = 0.806).

The age‐stratified dose‐response curves revealed distinct patterns: In young adults, HbA1c remained relatively stable across fat intake levels, with a modest increase only at extremes; middle‐aged adults showed a pronounced U‐shaped curve, with HbA1c decreasing to a nadir at 36.9% and then increasing steeply at higher intakes; older adults exhibited a shallower U‐shape, with the lowest HbA1c observed at 32.7% and a gradual rise in glycemic levels with increasing fat consumption.

### 3.5. Subgroup Analysis of Optimal Fat Intake by Diabetes Status

**Figure 4:**
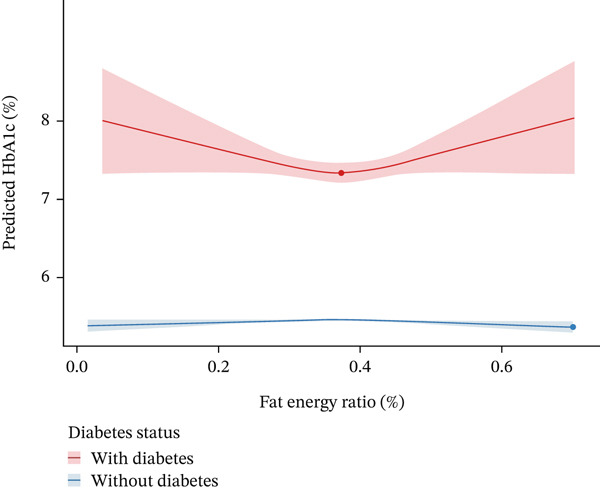
Association between dietary fat energy ratio and HbA1c stratified by diabetes status.

Subgroup analysis stratified by diabetes status was conducted to characterize the association between fat energy ratio and predicted HbA1c (Figure 4). For participants with diabetes (*n* = 1248, red curve), the relationship exhibited a statistically significant nonlinear pattern (*p* < 0.001 for nonlinearity), with a distinct U‐shaped trajectory. By solving the first derivative of the fitted curve, the optimal fat energy ratio was estimated to be 38.0% (95% CI: 35.7%–40.2%), at which the predicted HbA1c reached a nadir of 7.34% (SE = 0.15). This nadir was validated via bootstrap resampling (1000 iterations), yielding a stable median estimate with minimal variability (SD = 1.1*%*).

In contrast, participants without diabetes (*n* = 5391, blue curve) showed a flat dose‐response relationship, with no significant nonlinearity (*p* = 0.68). Although the fully adjusted linear model (Model 3, Table 2) indicated a statistically significant positive association in this subgroup, the RCS model revealed that this association was linear in form and clinically negligible in magnitude. Consequently, predicted HbA1c remained relatively stable across the entire range of fat energy ratios, showing minimal fluctuation regardless of dietary fat proportion, averaging 5.37% (SE = 0.08).

## 4. Discussion

### 4.1. Principal Findings and Mechanistic Interpretations

This large‐scale epidemiological investigation, leveraging nationally representative data from NHANES 2017–2020, delineates a significant nonlinear, U‐shaped relationship between dietary fat intake and glycemic control. The identification of an optimal fat energy ratio of 36.0%, corresponding to the nadir of predicted HbA1c, challenges the conventional linear paradigm and underscores a critical balance in macronutrient composition for metabolic health. The steepness of the curve′s right limb (high fat intake) was notably more pronounced than the left (low fat intake), suggesting that the detrimental glycemic effects of excessive fat consumption may be more acute than those of fat restriction.

The elevated HbA1c observed at low fat intakes (Q1: 26.6%) likely represents a proxy for high carbohydrate consumption, as evidenced by the reciprocal and significantly higher carbohydrate energy contribution (54.9%) in this quartile. This finding is physiologically plausible; diets high in carbohydrates, particularly refined and high‐glycemic‐index varieties, can induce recurrent postprandial hyperglycemia and increase hepatic de novo lipogenesis, thereby exacerbating insulin resistance over time [14–16]. Our results resonate with controlled feeding trials, which demonstrate that substituting carbohydrates for saturated fat yields no net benefit for insulin sensitivity and may worsen atherogenic dyslipidemia—a hallmark of the metabolic syndrome [17].

Conversely, the sharp increase in HbA1c at very high fat intakes (Q4: 46.1%) can be attributed to a confluence of mechanisms. Chronic consumption of energy‐dense, high‐fat diets, particularly those rich in saturated fatty acids, has been shown to activate innate immune pathways (e.g., Toll‐like Receptor 4 signaling), promote ectopic lipid deposition in liver and muscle, and induce mitochondrial overload and endoplasmic reticulum stress. These cellular disturbances collectively impair insulin signal transduction and promote hepatic gluconeogenesis [18–20]. Furthermore, diets at this extreme often displace fruits, vegetables, and whole grains, leading to a reduced intake of dietary fiber, magnesium, and polyphenols—micronutrients with established roles in improving insulin sensitivity and mitigating oxidative stress [21–23]. The significant positive association between fat intake and BMI in our baseline data further suggests that energy overconsumption, facilitated by the high palatability and energy density of fatty foods, may be a mediating factor.

Notably, the slight divergence between the primary RCS and supplementary quadratic regression results (1.2%–1.5% absolute difference in turning points) is ascribed to their intrinsic methodological characteristics. The RCS method, chosen as the primary approach, flexibly accommodates the asymmetric U‐shaped relationship observed in the data (with a steeper right limb), which aligns with the physiological reality of fat metabolism—where excessive fat intake exerts more acute detrimental effects on glycemic control than fat restriction. In contrast, quadratic regression inherently imposes a symmetric curve, a fundamental limitation that renders it less adept at capturing complex biological nonlinearity. Consequently, the RCS‐derived turning points (36.0% in the total population and 38.0% in participants with diabetes) remain the primary optimal estimates for clinical translation and interpretation, whereas the quadratic regression results provide complementary evidence to support the robustness of the primary findings.

### 4.2. Age as an Effect Modifier: A Lifespan Perspective

A pivotal and novel contribution of our study is the elucidation of age‐specific optimal fat intakes. The significantly higher optimal ratio of 36.9% for middle‐aged adults (45–64 years) compared with their younger (32.2%) and older (32.7%) counterparts reflects distinct pathophysiological and nutritional priorities across the lifespan.

Middle adulthood is characterized by a high prevalence of weight gain, a decline in lean muscle mass, and an increasing incidence of insulin resistance [24, 25]. In this context, a moderate increase in dietary fat at the expense of refined carbohydrates may enhance satiety, reduce between‐meal snacking, and provide a more stable energy substrate, thereby mitigating the large glycemic fluctuations associated with high‐carbohydrate meals [26, 27]. The pronounced U‐shaped curve in this group underscores their heightened metabolic vulnerability to macronutrient imbalances.

In contrast, the convergence of optimal ratios at 32.2% and 32.7% for younger and older adults, respectively, suggests different underlying rationales. For older adults (≥ 65 years), the primary nutritional challenge often shifts from managing energy surplus to preventing sarcopenia and malnutrition. A lower optimal fat intake may be indicative of a greater need to allocate daily energy quotas to high‐quality protein and micronutrient‐dense carbohydrates to support musculoskeletal health and immune function [28, 29]. The shallower U‐shaped curve in this demographic implies a broader tolerance range, but a preference for moderation to avoid unnecessary metabolic burden.

### 4.3. Clinical Implications for Diabetes Management

The stark divergence in the fat‐HbA1c relationship by diabetes status carries profound implications for clinical nutrition therapy. The clear, significant U‐shaped curve in individuals with diabetes (optimal: 38.0%) affirms that glycemic control in this population is exquisitely sensitive to dietary fat composition. This supports the principle of carbohydrate moderation, a cornerstone of many medical nutrition therapy approaches for diabetes [28, 30, 31]. It is critical to note, however, that even at its optimal point, the predicted HbA1c for diabetics was 7.34%, emphasizing that dietary fat adjustment is a complementary strategy, not a replacement for pharmacotherapy or overall dietary quality.

For the large nondiabetic population, the dose‐response relationship between dietary fat and HbA1c was predominantly flat, as revealed by the RCS model. Although the fully adjusted linear model indicated a statistically significant association, the nonlinear analysis demonstrated that the actual change in HbA1c across a wide fat intake range (approximately 25%–45% of energy) was minimal and clinically negligible. This indicates that, in the context of a functioning metabolic system, the proportion of dietary fat has a limited impact on medium‐term glycemic levels, offering substantial flexibility for public health guidelines and individual dietary personalization based on food preferences and cultural practices, without undue concern for glycemic consequences.

### 4.4. Public Health Relevance and Future Directions

Against the backdrop of vigorous public debates on optimal dietary patterns—largely polarized between extreme low‐fat and high‐fat (e.g., ketogenic) dietary paradigms—our findings offer population‐based evidence. This study empirically challenges the adoption of such nutritional extremes for maintaining general glycemic health.

Notably, across diverse subgroups, the identified optimal fat intake values fell within a range of approximately 32% to 38% of total energy. Although this range overlaps with the upper margin of the current acceptable macronutrient distribution range (AMDR) for fat (20%–35%) [12], our subgroup analyses revealed substantial heterogeneity in optimal intake thresholds. For instance, the ideal proportion was observed to vary meaningfully with factors such as age and diabetes status, indicating that a uniform fat intake target may be unsuitable for metabolically diverse populations.

These results prompt a reexploration of current dietary guidelines from multiple dimensions. In practical terms, healthcare providers can leverage this evidence to develop patient‐tailored dietary interventions. For example, in primary care settings, clinicians could use these findings to guide patients with metabolic syndrome toward carbohydrate intakes within the identified optimal range of 32%–38%, prioritizing high‐quality carbohydrates (e.g., whole grains, legumes, and vegetables) over refined sources, as the quality of carbohydrates has been shown to significantly impact cardiovascular risk [32–34]. This approach could potentially reduce the risk of hyperglycemia and associated complications. Moreover, these results hold implications for public health initiatives. Public health messaging could shift from debating macronutrient extremes to emphasizing a balanced, moderate carbohydrate intake tailored to individual needs and promoting carbohydrate quality.

Future studies could focus on elucidating the biological mechanisms underlying the observed differences in optimal carbohydrate intake across subgroups. Additionally, randomized controlled trials could explore how sustained adherence to diets within the identified range impacts long‐term health outcomes, such as cardiovascular disease risk and all‐cause mortality. By bridging the gap between research and practice, these subsequent investigations can further refine our understanding of personalized nutrition and inform evidence‐based dietary recommendations, ultimately transcending the simplistic low‐fat versus high‐fat dichotomy.

### 4.5. Limitations

The interpretation of our findings should be considered in the context of the study′s limitations. First, due to the observational nature of our cross‐sectional design, the identified associations between fat intake and HbA1c cannot be established as causal, and the possibility of residual confounding persists despite our robust adjustment for known covariates. Second, although we employed the average of two 24‐h dietary recalls with survey weights to enhance accuracy, self‐reported dietary data are inherently subject to measurement errors and recall bias. Most importantly, our primary focus on total fat intake, whereas foundational, necessarily amalgamates the potentially divergent effects of specific fatty acid subtypes (e.g., the putative benefits of unsaturated fats versus the detrimental effects of trans fats). This scope underscores a critical avenue for future research to delineate the distinct physiological roles of these subtypes in glucoregulatory physiology.

## 5. Conclusion

In summary, this study establishes a significant U‐shaped relationship between dietary fat intake and HbA1c, identifying 36.0% as the overall optimal fat energy ratio for glycemic health. Crucially, this central estimate is not one‐size‐fits‐all. Our findings demonstrate that the ideal fat intake is modifiable, being significantly higher for middle‐aged adults (36.9%) and for individuals with diabetes (38.0%) compared with their younger and older counterparts (approximately 32%). This evidence challenges universal dietary extremes and provides an empirical foundation for transitioning from rigid nutritional prescriptions to a personalized framework for dietary guidance.

## Funding

No funding was received for this manuscript.

## Conflicts of Interest

The authors declare no conflicts of interest.

## Data Availability

The data that support the findings of this study are openly available in NHANES at (https://wwwn.cdc.gov/nchs/nhanes/continuousnhanes/default.aspx?Cycle=2017‐2020), Reference Number (2017‐2020).
